# Antibody-Based PET Imaging of Misfolded Superoxide Dismutase 1 in an Amyotrophic Lateral Sclerosis Mouse Model

**DOI:** 10.2967/jnumed.124.268343

**Published:** 2025-01

**Authors:** Jacques A. Rousseau, Marcel Maier, Samia Ait-Mohand, Véronique Dumulon-Perreault, Otman Sarrhini, Sébastien Tremblay, Etienne Rousseau, Michael Salzmann, Brigitte Guérin

**Affiliations:** 1 Department of Nuclear Medicine and Radiobiology, Faculty of Medicine and Health Sciences, Université de Sherbrooke, Sherbrooke, Québec, Canada;; 2 AL-S Pharma AG, Schlieren, Zurich, Switzerland; and; 3 Sherbrooke Molecular Imaging Center/Centre de Recherche du CHUS, Sherbrooke, Québec, Canada

**Keywords:** amyotrophic lateral sclerosis, misfolded superoxide dismutase 1, [^89^Zr]Zr-DFO-α-miSOD1, PET imaging, ALS transgenic mice

## Abstract

Amyotrophic lateral sclerosis (ALS) is a rare neurodegenerative disease characterized by motor neuron loss in the motor cortex, brain stem, and spinal cord. Mutations in the superoxide dismutase 1 (SOD1) gene, resulting in misfolding of its protein product, are a common cause of ALS. Currently, there is no approved ALS diagnostic tool. Here, we present the development of a PET radiotracer, [^89^Zr]Zr-desferoxamine (DFO)-α-miSOD1, targeting selectively misfolded SOD1 (misSOD1). **Methods:** DFO-α-miSOD1 was prepared by conjugating α-miSOD1 antibody with DFO and labeled with ^89^Zr. A longitudinal imaging study was performed to identify the optimal mouse age and time after administration of [^89^Zr]Zr-DFO-α-miSOD1 for the detection of misSOD1 aggregation in transgenic mice overexpressing misSOD1 and in wild-type mice. Subsets of mice were either coinjected with an excess of α-miSOD1 or imaged with deglycosylated [^89^Zr]Zr-DFO-α-miSOD1 to assess target specificity. The internal radiation dose for [^89^Zr]Zr-DFO-α-miSOD1 was estimated by extrapolating data from mouse biodistribution experiments. **Results:** Imaging with [^89^Zr]Zr-DFO-α-miSOD1 was optimal in 136-d-old transgenic mice on day 10 after administration. Significant accumulation of [^89^Zr]Zr-DFO-α-miSOD1 was detected in the spinal cord and cartilage of ALS transgenic mice compared with the wild-type mice (*P* = 0.01). The radiotracer accumulation is selective and blockable with an excess of α-miSOD1. Deglycosylated [^89^Zr]Zr-DFO-α-miSOD1 results in high-contrast detection of misSOD1 but is prone to aggregation. The dosimetry for [^89^Zr]Zr-DFO-α-miSOD1 is comparable to that for other ^89^Zr-based tracers currently used in humans. **Conclusion:** This work thus establishes that [^89^Zr]Zr-DFO-α-miSOD1 PET can detect misSOD1 in transgenic mice, paving the way for application in early diagnosis of ALS and therapeutic monitoring.

## Visual Abstract

**Figure:**
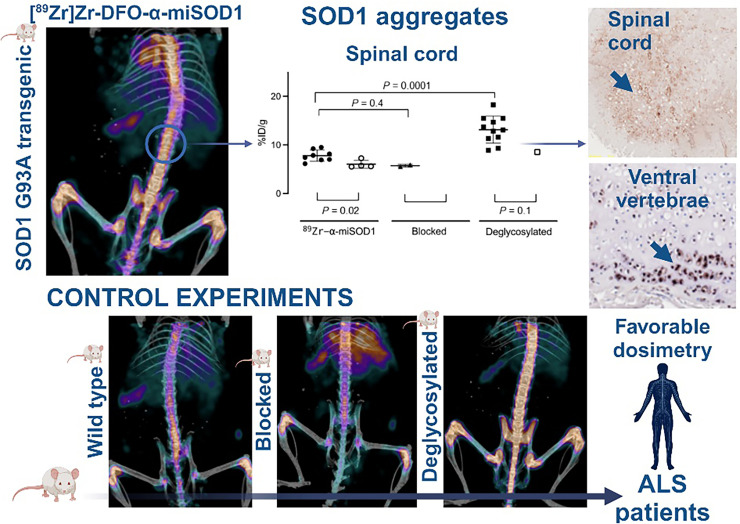

Amyotrophic lateral sclerosis (ALS) is a rare, progressive, and fatal neurodegenerative disease characterized by adult-onset loss of motor neurons in the motor cortex, brain stem, and spinal cord (1). The typically rapid decline in neurologic function results from the death of motor neurons that enable movement, speech, swallowing, and eventually breathing (2). Average life expectancy for people with ALS is 3–5 y from the time of symptom onset (3).

Of all ALS cases, 5%–10% have a genetic form and the rest are sporadic ALS. Superoxide dismutase 1 (SOD1) is one of multiple genes that have been implicated in ALS (4). SOD1 is a ubiquitously expressed antioxidant enzyme with important cellular functions in the detoxification of superoxide anion radicals (5). Most mutations in the SOD1 gene lead to the misfolding of its protein product and are the second most common genetic cause of ALS (4*,*^6^). The exact mechanism of mutant-SOD1 toxicity is still unknown. Most evidence points to a pathologic gain of function associated with the propensity of this protein to misfold and aggregate (7–11). Detection of misfolded wild-type (Wt) SOD1 within human postmortem sporadic ALS samples using misfolded SOD1 (misSOD1)-selective antibodies has been used to support the hypothesis that misfolded forms of Wt SOD1 can contribute to sporadic ALS pathogenesis (8–10*,*^12^–^17^).

We showed that the human-derived antibody α-miSOD1, also called AP-101, displays selective high-affinity binding to misSOD1 but not to physiologic SOD1 dimers (14). α-miSOD1 also binds to aggregated SOD1 in neurons of SOD1 mutation carriers with ALS and identified misSOD1 in human postmortem spinal cord tissue sections from a large fraction of patients with sporadic ALS (14). Administered either peripherally or directly to the central nervous system, a chimeric version of α-miSOD1 rescued motor deficits, attenuated loss of spinal cord motor neurons, and prolonged overall survival in 3 different mouse models of ALS (14). Clinical studies have been initiated to investigate the safety and disease-modifying potential of this antibody in ALS patients (https://www.clinicaltrials.gov/study/NCT05039099).

Currently, there is no approved diagnostic tool for ALS (18). An imaging test for early detection of ALS and for monitoring disease progression would have significant diagnostic and prognostic value. PET is a noninvasive and quantitative imaging technique that has proven to be highly valuable for the study of neurodegenerative diseases (19). In this work, we explored the potential of α-miSOD1 conjugated with desferoxamine (DFO) and labeled with ^89^Zr (half-life, 78.41 h) for the in vivo detection of misSOD1. We first characterized the PET tracer and conducted a longitudinal imaging study to determine the optimal age of mice and the best time after administration of [^89^Zr]Zr-DFO-α-miSOD1 for detecting misSOD1 aggregation in transgenic mice expressing misSOD1, using Wt mice as controls. Subsets of mice were coinjected with an excess of α-miSOD1 or imaged with deglycosylated [^89^Zr]Zr-DFO-α-miSOD1 (20) to assess target specificity. The dosimetry of this radiopharmaceutical was examined for its human use.

## MATERIALS AND METHODS

Details on the experimental procedures and analytic methods are provided in the supplemental materials available at http://jnm.snmjournals.org.

### Preparation of DFO-α-miSOD Conjugates

The synthesis of DFO-α-miSOD1 conjugates is described in the supplemental materials. The average number of DFOs per molecule of α-miSOD1 was determined using an isotopic dilution assay (21). The concentration of the conjugate was measured spectrophotometrically using a protein assay (22).

### Radiolabeling of DFO-α-miSOD Conjugates

[^89^Zr]ZrCl_4_ was prepared using a modified method developed by Pandya et al. (23) using ^89^Y-pressed targets (24) as described in the supplemental materials. The radiolabeling yield was assessed by radio–thin-layer chromatography using 50 mM diethylenetriaminepentaacetic acid (pH, 5.0–6.0) as the mobile phase and counted on a thin-layer chromatography plate reader. Radiochemical purity was determined using radio–thin-layer chromatography and sodium dodecyl–sulfate polyacrylamide gel electrophoresis as described the supplemental materials. The specific activity was determined following our previous reported procedure (24).

### Plasma Stability

^89^Zr-radiotracers (175–200 MBq) in phosphate-buffered saline were incubated at 37°C in 500 μL of fresh mouse plasma (1/1). The stability was directly monitored by radio–thin-layer chromatography using 50 mM diethylenetriaminepentaacetic acid as a mobile phase to follow the presence of free ^89^Zr (24) over a 10-d period after radiolabeling.

### Animals

Animal studies complied with the Université de Sherbrooke Institutional Animal Care and Use Committee (protocol 2017-2118) and the Canadian Council on Animal Care guidelines. Tissue collection of SOD1^G37R^ mice was approved by the veterinarian authorities of the Canton of Zurich (animal license 82-2014) in compliance with Swiss law (“455.163 Tierversuchsverordnung,” 2010). Female SOD1^G93A^ and SOD1^G37R^ transgenic mice expressing the ALS genotype, as well as Wt mice (C56BL/6J), were purchased from The Jackson Laboratory. SOD1^G37R^ mice overexpressing human SOD1 with a G37R mutation have a high propensity to form misSOD1 aggregates in the spinal cord and brain (25).

### PET Imaging

Radiotracer administration and PET imaging were done under isoflurane anesthesia (induction, 2%–3% isoflurane, and then 1.0%–2.5%; 1.0–1.5 L of oxygen/min). [^89^Zr]Zr-DFO-α-miSOD1 or deglycosylated [^89^Zr]Zr-DFO-α-miSOD1 (20–30 MBq/0.2 mL 0.9% saline) was administrated via a catheter (30-gauge needle/polyethylene-10 tube) installed within the tail vein. For the blocking studies, the radiotracer was coinjected with a 100 mg/kg dose of α-miSOD1. Imaging was done on a LabPET-II PET scanner (IR&T). The mouse was positioned within the field of view of the PET imager, and a 30-min static acquisition was performed. The bed of the imager was then moved within the CT module of the LabPET8 scanner, and a 2-min CT acquisition was performed. Throughout PET imaging, the mouse was kept warm using pulsed hot air maintained at 32°C and the environmental temperature and respiration rate were monitored (PCSAM, model 1025T monitoring and gating system; SAII). Details on image processing and analysis are described in the supplemental materials. The overall ratio of spinal cord to vertebra was quantified by tracing a thin longitudinal region of interest (ROI) going through the length of these tissues (Supplemental Fig. 1A). Further volumetric ROIs were drawn on the head of the femur and spleen (Supplemental Figs. 1B and 1C). Because of the radiotracer within the ventral vertebra, the ROI traced on the spinal cord was corrected for the partial-volume effect according to the following equation: corrected activity = (measured activity − spill-in fraction)/recovery coefficient (26). The recovery coefficient and spill-in fraction were obtained using the Ultra-Micro Hot Spot phantom (DataSpectrum) (27).

### MisSOD1 Aggregate Identification

Potential misSOD1 aggregates appear as discrete radiotracer spots within the spinal cord. A spot was considered valid only if it was well within the spinal cord and not contaminated by radioactivity spilling in from the vertebra. For each valid spot, a volumetric ROI was drawn to include the whole misSOD1 aggregate. The aggregate uptakes were expressed in terms of percentage injected dose per gram (%ID/g). In converting Bq/mL to %ID/g, we assumed the tissue density was equal to 1 g/mL. The misSOD1 aggregate uptake was compared with %ID/g values measured in a similar ROI drawn within the same mouse spinal cord (internal reference) and to a similar ROI traced in the same location of the spinal cord of Wt mice from the same group (external reference).

### Perfusion and Biodistribution

After the last PET imaging procedure on day 10, the mice were euthanized by CO_2_ inhalation under isoflurane anesthesia and the main tissues were sampled to measure their radiotracer content as described in the supplemental materials. The spinal cords were fixed in 4% paraformaldehyde for 24 h for histopathologic analysis.

### Statistical Analysis

A 1-way ANOVA Dunnett comparison test was used to assess the impact of blocking and deglycosylation on the uptake of [^89^Zr]Zr-DFO-α-miSOD1 in transgenic and Wt mice. Within each experimental condition, the transgenic mice were compared with the Wt mice using an unpaired 2-tailed Student *t* test and 4 levels of significance: *P* < 0.05, 0.01, 0.001, and 0.0001.

### Dosimetry

Female and male Wt mice (*n* = 4 per time point) were injected through the caudal vein with [^89^Zr]Zr-DFO-α-miSOD1 (0.84–0.86 MBq) and euthanized at 2 h and on days 1, 3, 7, and 10 after injection. Non–decay-corrected biodistribution data were used to generate time–activity curves for [^89^Zr]Zr-DFO-α-miSOD1. OLINDA software (version 2.2.3; Hermes Medical Solutions) was used to estimate the dosimetry as described in the supplemental materials.

### Histology

Perfused and fixed whole vertebral column or bone of the hindlegs of untreated 4-mo-old SOD1^G93A^, 8-mo-old SOD1^G37R^ (25), or age-matched Wt mice was incubated for 5 d in 0.5 M EDTA, pH 7.4 (Sigma), at 37°C with mechanical agitation before paraffin embedding and sectioning to a thickness of 6 μm. All sections were deparaffinized, pretreated by cooking in citrate buffer followed by incubation for 10 min in concentrated formic acid, inactivated using peroxidase in 3% H_2_O_2_ in methanol, blocked in 5% horse serum/5% goat serum/4% bovine serum albumin in phosphate-buffered saline, and stained with 2 nM α-miSOD1 according to Maier et al. (14). Details on the detection of misSOD1 in sections of whole vertebral column and bones are described in the supplemental materials.

## RESULTS

### Synthesis and Characterization

The misSOD1-selective human antibody α-miSOD1 (14) was conjugated with DFO to obtain a DFO–to–α-miSOD1 ratio of 1.1:1.0 (*n = 7*) estimated by isotopic dilution techniques (21). A deglycosylated analog was prepared using EndoS2 endoglycosidase for the specific hydrolysis of Fc-glycans on the DFO−α-miSOD1 (20) to offer a DFO–to–deglycosylated-α-miSOD1 ratio of 0.6:1.0 (*n = 4*). The purity of the 2 DFO conjugates was confirmed by high-performance liquid chromatography analysis showing narrow peaks for the conjugates (retention time of 9.66 vs. 9.43 min for DFO-α-miSOD1 and deglycosylated-analog, respectively), and absence of aggregates was confirmed by sodium dodecyl–sulfate polyacrylamide gel electrophoresis analysis (Supplemental Figs. 2A and 2B). Natural Zr (^nat^Zr)-DFO-α-miSOD1 and the deglycosylated analog maintained a low nanomolar affinity (half-maximal effective concentration, 0.3 and 2.4 nM, respectively) and selectivity for misSOD1, like that of α-miSOD1 (half-maximal effective concentration, 0.2 nM; Supplemental Figs. 2C and 2D).

The DFO conjugates were subsequently labeled with ^89^Zr with a radiolabeling yield more than 95% (Supplemental Fig. 2E). [^89^Zr]Zr-DFO-α-miSOD1 is stable in plasma for up to 10 d (Supplemental Fig. 2F). The apparent specific activity of [^89^Zr]Zr-DFO-α-miSOD1 was twice as high as that of the deglycosylated analog (0.66 ± 0.11 GBq/mg vs. 0.32 ± 0.05 GBq/mg), consistent with its higher DFO–to–α-miSOD1 ratio. The DFO conjugation occurs mainly on the heavy chain of α-miSOD1 (Supplemental Fig. 2G). Direct deglycosylation of α-miSOD1 led to the formation of aggregates (Supplemental Fig. 2H). Stability analysis revealed that the deglycosylated [^89^Zr]Zr-DFO-α-miSOD1 is more prone to aggregation after its preparation.

### In Vivo PET Imaging

A longitudinal study was performed first to establish the best mouse age and the best time after radiotracer administration for PET imaging (Supplemental Table 1). Whole-body PET images showed the presence of [^89^Zr]Zr-DFO-α-miSOD1 in the ventral portion of the vertebra and in the spleen and the head of the femur (Supplemental Fig. 3). The accumulation of [^89^Zr]Zr-DFO-α-miSOD1, measured in %ID/g, was lower in the background and showed better contrast in 136-d-old transgenic mice than in Wt mice (Supplemental Fig. 3). By day 136, ALS symptoms were mild in 7% of mice, moderate in 65%, and severe in 28% (Supplemental Table 2).

**FIGURE 1.**
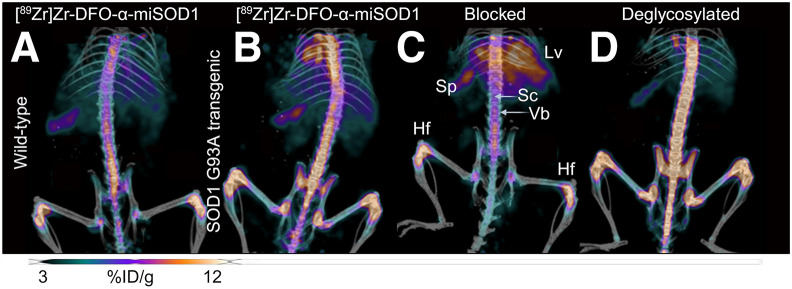
Whole-body maximal-intensity projection PET/CT images showing biodistribution of [^89^Zr]Zr-DFO-α-miSOD1 on day 10 after injection in 136-d-old Wt mice (A) and SOD1^G93A^ mice (B and C) under unblocked (B) and blocked conditions (C). (D) Biodistribution of deglycosylated [^89^Zr]Zr-DFO-α-miSOD1 in transgenic mice. Hf = head of femur; Lv = liver; Sc = spinal cord; Sp= spleen; Vb = ventral vertebra bone.

**FIGURE 2.**
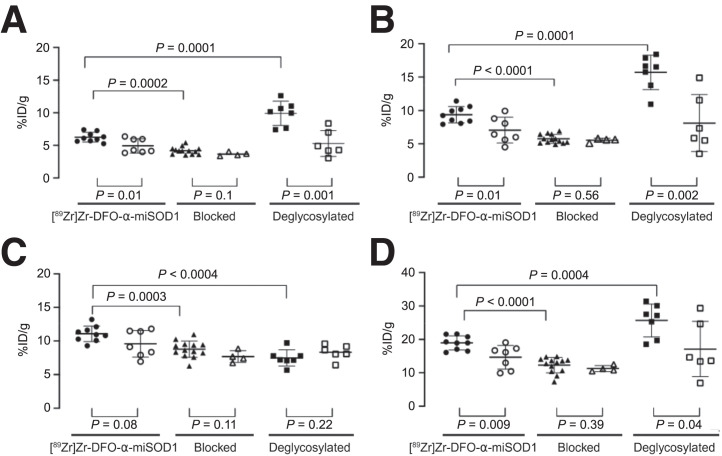
Uptake of [^89^Zr]Zr-DFO-α-miSOD1 in spinal cord (A), vertebra (B), spleen (C), and head of femur (D) in normal, blocked, and deglycosylated conditions. Experiment was conducted on 136-d-old SOD1^G93A^ mice (solid symbols) and their Wt controls (open symbols).

The accumulation of [^89^Zr]Zr-DFO-α-miSOD1 in transgenic mice was most important in the head of the femur, followed by the spleen, the ventral vertebra, and the spinal cord (Figs. 1 and 2). In all tissues, the accumulation of [^89^Zr]Zr-DFO-α-miSOD1 in transgenic mice exceeded that in Wt mice. Blocking with an excess dose of α-miSOD1 significantly decreased uptake of [^89^Zr]Zr-DFO-α-miSOD1 in the spinal cord, vertebra, head of the femur, and spleen, suggesting selective target binding in these tissues. Deglycosylated [^89^Zr]Zr-DFO-α-miSOD1 significantly increased uptake in the spinal cord, vertebra, and head of the femur while decreasing accumulation in the spleen. Moreover, blocking and deglycosylation did not significantly affect the accumulation of [^89^Zr]Zr-DFO-α-miSOD1 in the tissues of Wt mice.

### Quantification of PET-Positive misSOD1 Aggregates in the Spinal Cord

**FIGURE 3.**
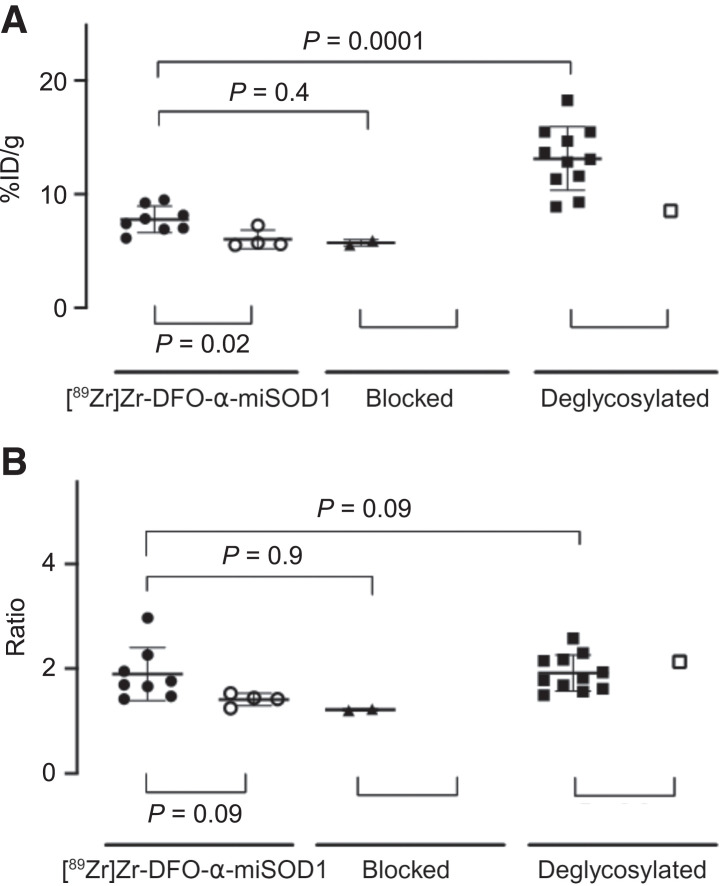
Quantification of radiotracer uptake in misSOD1 aggregates (%ID/g) (A) and ratio of misSOD1 aggregate to internal spinal cord reference (B) for transgenic (solid symbols) and Wt mice (open symbols) with [^89^Zr]Zr-DFO-α-miSOD1, blocked, or deglycosylated analogs.

A detailed analysis of the PET images revealed [^89^Zr]Zr-DFO-α-miSOD1 accumulation in misSOD1 aggregates within the spinal cord. The number of aggregates detected by [^89^Zr]Zr-DFO-α-miSOD1 and clearly separated from the vertebra bone signal (Supplemental Fig. 4) was more reliable and higher on day 10 than on day 7 after radiotracer injection (Supplemental Tables 3 and 4). Furthermore, all spinal cord aggregates that were identified with [^89^Zr]Zr-DFO-α-miSOD1 and its deglycosylated analog were found between the T11 and L2 vertebrae, which correspond to the L1 to L6 spinal cord segments (Supplemental Fig. 4) (28). Coinjection with an excess of α-miSOD1 considerably reduced the number of aggregates detected by [^89^Zr]Zr-DFO-α-miSOD1 (from 8 to 2), the percentage of mice having aggregate uptake (from 67% [6/9] to 15% [2/13]), and the intensity of the remaining aggregate uptake (from 8.1 ± 1.3 %ID/g to 5.7 ± 0.3 %ID/g; Fig. 3A; Supplemental Tables 3 and 5). The percentage of mice having aggregate uptake increased from 67% with [^89^Zr]Zr-DFO-α-miSOD1 to 100% with deglycosylated [^89^Zr]Zr-DFO-α-miSOD1, with an approximately 60% increase in signal intensity for deglycosylated [^89^Zr]Zr-DFO-α-miSOD1 (Fig. 3A, Supplemental Tables 3 and 6), whereas the ratio of aggregate to internal spinal cord reference remained similar for both radiotracers (Fig. 3B). PET signals were also detected in the spinal cord area of Wt mice with both radiotracers, but with a significantly lower frequency and signal intensity, providing good imaging contrast between transgenic and Wt mice (Fig. 3A; Supplemental Tables 3, 7, 8, and 9).

### Ex Vivo Biodistribution in Organs

**FIGURE 4.**
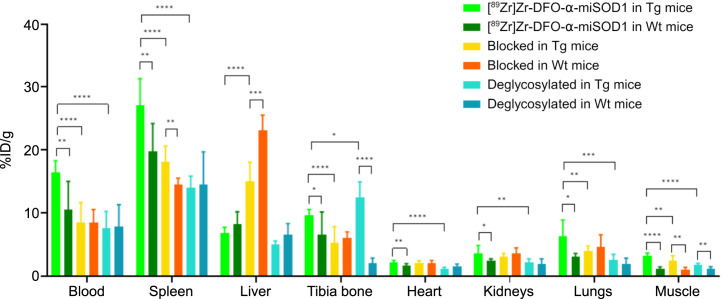
Residual radioactivity (%ID/g) of main nontarget tissue 10 d after injection of [^89^Zr]Zr-DFO-α-miSOD1, after blocking, or after deglycosylation in transgenic and Wt mice measured in total blood or after perfusion in spleen, liver, tibia bone, heart, kidneys, lungs, and muscle. Tg = transgenic.

After the last PET acquisition on day 10, the mice were dissected and residual activity in blood and tissues of interest was measured (Fig. 4). Radiotracer accumulation was lower in the blood for blocked and deglycosylated [^89^Zr]Zr-DFO-α-miSOD1 than for injected [^89^Zr]Zr-DFO-α-miSOD1: 8.49 ± 3.12 %ID/g and 7.56 ± 2.65 %ID/g versus 16.38 ± 1.90 %ID/g (Fig. 4; Supplemental Tables 10–12). Consistent with in vivo signal quantification by PET imaging, a higher bone accumulation (12.47 ± 2.45 %ID/g vs. 9.60 ± 0.92 %ID/g) and a lower residual activity in spleen (13.96 ± 1.87 %ID/g vs. 27.07 ± 4.25 %ID/g) were observed for deglycosylated [^89^Zr]Zr-DFO-α-miSOD1 than for injected [^89^Zr]Zr-DFO-α-miSOD1 (Fig. 4; Supplemental Tables 10 and 12). [^89^Zr]Zr-DFO-α-miSOD1 showed lower accumulation in the blood and most tissues of Wt mice except the liver (Fig. 4; Supplemental Tables 10, 11, 13, and 14). A similar biodistribution pattern was observed for deglycosylated [^89^Zr]Zr-DFO-α-miSOD1 in Wt mice (Fig. 4; Supplemental Tables 12 and 15).

### Accumulation of misSOD1 in Spinal Cord and Cartilage Tissue of SOD1^G93A^ and SOD1^G37R^ Transgenic Mice

**FIGURE 5.**
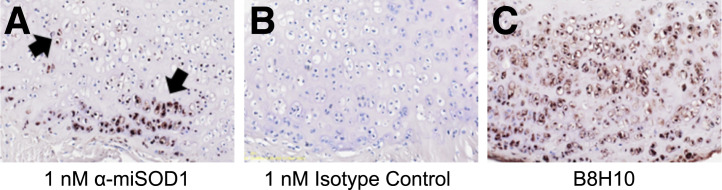
Localization of misSOD1 accumulations in cartilage tissue of ventral vertebrae bone of 4-mo-old SOD1^G93A^ mice using immunohistochemistry with α-miSOD1. (A) α-miSOD1–positive accumulation was identified in chondrocytes (arrows) of cartilage tissue on transaxial histologic sections through ventral vertebra segment. (B) No immunoreactivity was detected using isotype control antibody on adjacent sections of same tissues, confirming specificity of staining to misSOD1. (C) Immunoreactivity for α-miSOD1 overlapped with staining using different misSOD1 antibody B8H10 on adjacent sections.

After dissection, the spinal cord, cartilage tissue of the femur, and dorsal and ventral vertebra segments were extracted to analyze by immunohistochemistry the accumulation of misSOD1. ^nat^Zr-DFO-α-miSOD1 and deglycosylated and nonmodified α-miSOD1 detected misSOD1 in a similar manner on spinal cord sections of a SOD1^G93A^ mouse (Supplemental Fig. 5). We also investigated the presence of misSOD1 in cartilage tissue of 2 ALS animal models, SOD1^G93A^ and SOD1^G37R^ mice, using immunohistochemistry. MisSOD1 is detected in chondrocytes of cartilage in the vertebrae and head of the femur of 4-mo-old SOD1^G93A^ and 8-mo-old SOD1^G37R^ mice (Fig. 5A; Supplemental Figs. 6A, 6B, 7A, 7B, and 7C) (27), whereas no signal was observed using an isotype control antibody (Fig. 5B; Supplemental Figs. 6C, 6D, 7D, 7E, and 7F) or a ubiquitous staining of tissue when a total SOD1 antibody, Abcam 79,390, was used as a detection antibody. The immunoreactivity of α-miSOD1 overlapped with staining obtained using another misSOD1 antibody, B8H10, on adjacent sections, confirming the presence of misSOD1 in the cartilage tissue of vertebrae and in the head of the femur of SOD1^G93A^ and SOD1^G37R^ mice (Fig. 5C; Supplemental Figs. 6E, 6F, 7G, 7H).

### Dosimetry

To estimate the dosimetry of [^89^Zr]Zr-DFO-α-miSOD1, a biodistribution experiment was conducted at 2 h and on days 1, 3, 7, and 10 after injection using 126-d-old female and male Wt mice. The biodistribution data are presented in Supplemental Figure 8 and Supplemental Table 16. From the biodistribution results, uptake of [^89^Zr]Zr-DFO-α-miSOD1 was high in the lungs at 2 h after injection and rapidly decreased over time. The dosimetry was calculated from radiotracer residence time using the OLINDA software (version 2.2.3). The largest radiation doses were observed in the lungs (0.0535 vs. 0.0473 mSv/MBq) and liver (0.0515 vs. 0.0336 mSv/MBq) for female versus male mice, respectively. The estimated effective dose for [^89^Zr]Zr-DFO-α-miSOD1 is higher in female (0.237 mSv/MBq) than male (0.171 mSv/MBq) mice (Supplemental Table 17).

## DISCUSSION

A radiotracer for early detection of ALS and for monitoring disease progression could offer substantial benefits in terms of diagnosis and outcome prediction (29). In this work, we showed that ^89^Zr-DFO-α-miSOD1 detects accumulations of misSOD1 in the spinal cord and in the cartilage of the vertebrae and other joints in SOD1^G93A^ mice by PET imaging. Coinjection of an excess of α-miSOD1 displaces the radiotracer and considerably reduces its accumulation in these tissues, supporting the selectivity of [^89^Zr]Zr-DFO-α-miSOD1 and the potential low contribution of free ^89^Zr to bone uptake (30). When the interaction with Fc-γ-receptors via deglycosylation of [^89^Zr]Zr-DFO-α-miSOD1 was reduced (20), an improved in vivo performance was observed, resulting in decreased uptake in the healthy liver and spleen and increased uptake in tissues containing misSOD1. However, the deglycosylated [^89^Zr]Zr-DFO-α-miSOD1 is more prone to aggregation after its preparation, and this issue will have to be addressed in further investigations.

There was more prevalent [^89^Zr]Zr-DFO-α-miSOD1 PET detection of misSOD1 aggregates in the lumbar spinal cord in SOD1^G93A^ mice, and this correlates with the age-dependent accumulation of misSOD1 aggregates. The strong direct correlation of in vivo misSOD1 aggregate uptake with regions matched by immunohistology analyses of misSOD1 aggregates in the same mice supports the validity of using [^89^Zr]Zr-DFO-α-miSOD1 for misSOD1 aggregate PET imaging as a method for in vivo evaluation of misSOD1. Interestingly, few aggregates were observed in the spinal cords of Wt mice imaged with the 2 PET tracers, and none were detected in the blocked group. There is no clear explanation for these results; further investigations are needed.

A new finding of our study was detection of misSOD1 in vertebrae and other joints by [^89^Zr]Zr-DFO-α-miSOD1 PET in SOD1^G93A^ mice, as confirmed by immunohistology with α-miSOD1 and with other antibodies detecting misSOD1 in cartilage in 2 ALS animal models. Bone alterations in ALS mice are in line with others’ observations of impaired bone homeostasis and bone loss in ALS mice, severely impaired properties of osteoblasts in primary cultures and bones from SOD1^G93A^ mice relative to Wt mice (31), and abnormal mitochondrial dynamics in osteocytes (32). Provided that misSOD1 accumulation can be confirmed in bones of ALS patients, a contribution of misSOD1 to general ALS pathophysiology—which includes poor bone quality—should be considered (33).

The calculated effective dose of [^89^Zr]Zr-DFO-α-miSOD1 was greater in female than male mice. Effective doses are about 20%–40% higher for women than for men, a trend observed across various radiopharmaceuticals (34). The estimated effective doses of [^89^Zr]Zr-DFO-α-miSOD1 are acceptable when compared with other ^89^Zr-based tracers in use in humans. Indeed, the effective dose from the animal data for [^89^Zr]Zr-DFO-trastuzumab was calculated at 0.44 mSv/MBq in female animals and 0.39 mSv/MBq in male animals—a dose that was found to be safe for a phase 0–1 study (35). Although this value is higher than that of [^18^F]FDG PET, ^89^Zr-based PET tracers are safe for clinical use (35).

## CONCLUSION

Our study represents a substantial contribution to the ongoing efforts to develop a PET radiotracer for detecting misSOD1 aggregation in ALS, supporting the exploration of [^89^Zr]Zr-DFO-α-miSOD1 in ALS patients.

## DISCLOSURE

Brigitte Guérin is the holder of the Jeanne and J.-Louis Lévesque Chair in Radiobiology at Université de Sherbrooke and a member of the CRCHUS funded by the Fonds de Recherche du Québec–Santé. Etienne Rousseau is supported by a Fonds de Recherche du Québec Health clinical research scholar grant. This work was partially financed by the Ministère de l’Économie et de l’Innovation (PSOv2d-54218). One Patent Cooperation Treaty patent was filed for some of the material presented in this article. Marcel Maier and Michael Salzmann are employees of Neurimmune AG. No other potential conflict of interest relevant to this article was reported.

## Notes

KEY POINTS **QUESTION:** How does the development of [^89^Zr]Zr-DFO-α-miSOD1 facilitate the noninvasive detection of misSOD1 in ALS transgenic mice? **PERTINENT FINDINGS:** A robust synthesis of [^89^Zr]Zr-DFO-α-miSOD1 enabled imaging of misSOD1 aggregation in spinal cord and cartilage of ALS transgenic mice. We also confirmed that the calculated effective dose of [^89^Zr]Zr-DFO-α-miSOD1 is safe for clinical use. **IMPLICATIONS FOR PATIENT CARE:** [^89^Zr]Zr-DFO-α-miSOD1 offers high-contrast detection of misSOD1 with minimal uptake in healthy organs, making it a promising tracer for ALS patients.
